# Excitons in Epitaxially Grown WS_2_ on Graphene: A Nanometer-Resolved Electron Energy Loss Spectroscopy and Density Functional Theory Study

**DOI:** 10.1021/acsnano.5c11994

**Published:** 2025-12-11

**Authors:** Max Bergmann, Jürgen Belz, Oliver Maßmeyer, Badrosadat Ojaghi Dogahe, Robin Günkel, Johannes Glowatzki, Andreas Beyer, Ivan Solovev, Jens-Christian Drawer, Martin Esmann, Sergej Pasko, Simonas Krotkus, Michael Heuken, Stefan Wippermann, Kerstin Volz

**Affiliations:** ‡ Institute of Physics, 11233 Carl von Ossietzky University of Oldenburg, 26129 Oldenburg, Germany; § 60988 AIXTRON SE, 52134 Herzogenrath, Germany; △ mar.quest | Marburg Center for Quantum Materials and Sustainable Technologies, Philipps-Universitat Marburg, 35032 Marburg, Germany; ▽ Department of Physics, Philipps-Universitat Marburg, Hans Meerwein Str. 6, 35032 Marburg, Germany

**Keywords:** TMDs, excitons, 2D heterostructures, MOCVD, STEM, EELS, graphene

## Abstract

We investigate the excitonic properties of epitaxially grown WS_2_ monolayers, bilayers and multilayers on graphene using monochromatic electron energy loss spectroscopy (EELS) in a scanning transmission electron microscope. This material system is particularly attractive for optoelectronic applications, as direct growth from the gas phase offers a scalable route to wafer-sized heterostructures. The combination of nanometer-scale spatial resolution and high spectral quality in EELS allows for a detailed analysis of layer-dependent excitonic features. To complement the experimental results, we perform *ab initio* simulations based on density functional theory and the Bethe–Salpeter equation. The experimental spectra reveal a systematic redshift of both A and B excitons at the K-valley—centered near 2.0 and 2.4 eV, respectively—as the number of WS_2_ layers increases. While such redshifts are often attributed to dielectric screening, our *ab initio* calculations show that the dominant contribution arises from a subtle lattice mismatch between the lower and upper WS_2_ layers. We trace this mismatch to the heteroepitaxial alignment of the first WS_2_ layer to the graphene substrate during the growth process. Our results highlight how nanoscale structural distortions in epitaxial 2D materials can strongly influence key excitonic properties, even in the absence of intentional strain or alloying. By combining nanometer-scale electron spectroscopy with advanced theory, we establish a direct link between atomic structure and excitonic response in realistic, nonidealized heterostructures. These findings underscore the importance of microscopic interface effects in the design and scalable fabrication of exciton-based optoelectronic devices.

## Graphical abstract

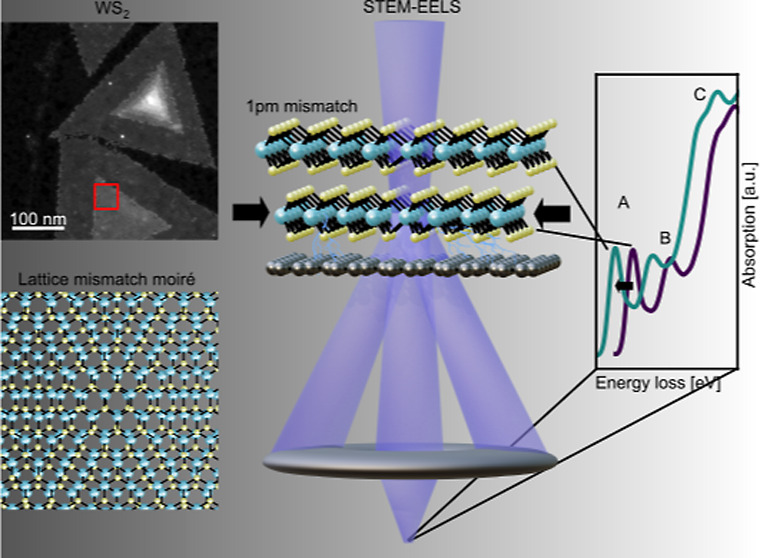

## Introduction

In the field of materials science, two-dimensional (2D) transition metal dichalcogenides (TMDs) have emerged as a captivating area of research with remarkable potential, particularly in the domain of valleytronics.^1–7^ TMDs form a diverse class of 2D materials, with semiconducting properties making them ideal for various applications like battery electrodes,^8^ solar cells^9,10^ and field-effect tunneling transistors.^11–13^ TMDs also feature an indirect-to-direct band gap transition in the monolayer (ML) limit, which leads to an enhanced quantum efficiency and significant increase in photoluminescence (PL),^14–16^ thus illustrating their potential for optoelectronic applications. The optoelectronic applications of these TMDs are fundamentally linked to the behavior of excitons at the *K*-point within the first Brillouin zone. A unique feature of TMDs’ electronic properties is their tunability through layer number variation. Additionally, TMDs such as WS_2_ possess spin–orbit interactions that couple the spin and valley degrees of freedom.

While hexagonal boron nitride (h-BN) is widely used as a substrate for 2D TMDs due to its atomically flat and inert surface, it typically requires mechanical transfer and is not easily scalable to wafer-sized heterostructures. In contrast, graphene offers several advantages: it supports direct epitaxial growth of WS_2_ from the gas phase with well-defined crystallographic alignment, a critical prerequisite for scalable device integration, especially it can even be used to tune the twist angle between TMD layers,^17^ which is known to strongly influence electronic properties.^18^ Moreover, graphene-induced strain fields can further modulate the local electronic structure, as explored in this work. Graphene is electrically conductive and compatible with electron energy loss spectroscopy in the scanning transmission electron microscope (STEM), enabling nanometer-scale spectral analysis without charging effects or beam-induced damage. Beyond characterization, graphene also offers practical advantages for device integration. Direct metal contacts to TMDs with gold or copper often lead to Schottky barriers and poor contact quality,^19^ whereas graphene can serve as a clean and tunable contact interface. Graphene itself can be produced on a large scale using copper substrates and chemical vapor deposition (CVD),^20,21^ enabling seamless integration with CVD-grown 2D TMDs into heterostructures. Graphene-contacted TMD devices—including field-effect tunneling transistors^13^ or memory devices^12^—have demonstrated improved performance compared to those with direct metal contacts.^22^ These examples demonstrate the technological relevance of understanding epitaxial WS_2_/graphene heterostructures, particularly with respect to their electronic and excitonic properties.

Excitons, quasiparticles consisting of electron–hole pairs bound by Coulomb-force, play an essential role in low-dimensional systems like ML TMDs.^23^ These systems are characterized by enhanced Coulomb interactions due to spatial confinement and reduced Coulomb screening.^24,25^ The existence of valley excitons in ML TMDs was originally predicted from theory.^26^ Their experimental observation has since been explored via techniques such as inelastic X-ray scattering,^27^ neutron scattering,^28^ and time- and angle-resolved photoelectron spectroscopy (trARPES).^29^ Electron energy loss spectroscopy (EELS),^18,30,31^ primarily conducted in monochromated scanning transmission electron microscopes (STEMs), stands out as a promising technique for investigating excitonic properties in ML TMDs, especially in conjunction with *ab initio* calculations, as explored in this work. STEM-EELS provides spatial resolution to visualize atomic structures, detects localized deviations, and correlates them with excitonic signals. Additionally, unlike phonon-assisted spectroscopic techniques, STEM-EELS enables indirect transitions through momentum transfer,^32^ leveraging the significantly higher momentum of electrons compared to photons. More recently, cathodoluminescence, which is directly accessible in a STEM, has also been shown to be an outstanding tool for investigating spectroscopic properties of excitons in correlation to the absorptive signal monitored by EELS.^33,34^

In this study, we employ STEM-EELS to investigate the energy shifts of excitons at the *K*-point that arise as a consequence of transitioning from a ML to a bi- or multilayer (BL, MuL) configuration, where the first layer of WS_2_ is strained as it has been fabricated in an epitaxial growth experiment from the gas phase. While previous studies focused on mechanically exfoliated moiré-twisted TMD systems,^18^ we investigate a stacked WS_2_ structure grown on graphene on a *c*-plane sapphire substrate *via* metal organic chemical vapor deposition (MOCVD). We show that the underlying graphene slightly reduces the lattice constant of the WS_2_ on top, inducing a lattice mismatch moiré-like distortion in the stacked bilayer region, which in turn affects the material’s excitonic properties. Our findings directly link the growth process of the sample to its electronic properties, which may differ from those observed in exfoliated samples. Several studies have explored excitonic excitations originating from the K–K gap^18,35–37^ and the associated spin–orbit coupling, known as the A and B excitons. However, none have examined the energy shifts of these excitations as a function of layer number and strain using EELS on a technologically highly relevant, namely grown heterostructure. Growth of TMDs from the gas phase is, however, highly relevant for device applications. Hence the real and electronic structure of these samples needs to be investigated in detail and correlated to each other. This work addresses this gap by comparing the energy shifts of *K*-point excitons with those of higher-energy excitonic effects residing at other points in the first Brillouin zone. First principle calculations, specifically density functional theory (DFT) with electron–hole interactions included *via* the Bethe–Salpeter equation (BSE),^38–41^ were used to interpret the spectra and explain the energy shifts.

## Results and Discussion

We first discuss the structure of the corresponding scanned sample region and the EELS data acquisition, followed by an evaluation of the EEL spectra in comparison with simulation results.

Figure 1a shows two WS_2_ flakes and is generated through high angle annular dark-field (HAADF) imaging, showing increased brightness with increasing number of layers allowing to count the number of WS_2_ layers. Next to as well as underneath the WS_2_ flakes we find the diffractions of graphene, as shown in Figure 1b. This confirms the heteroepitaxial alignment of the WS_2_ on the graphene achieved during MOCVD growth.^17^ The alignment is further confirmed by the NBD data in Figure S1, which shows that at the center of the flake, where nucleation during growth is likely initiated, the flake is aligned, and subsequently overgrows graphene layers with varying orientations. Further information can be found in Supplementary part I.

**Figure 1:**
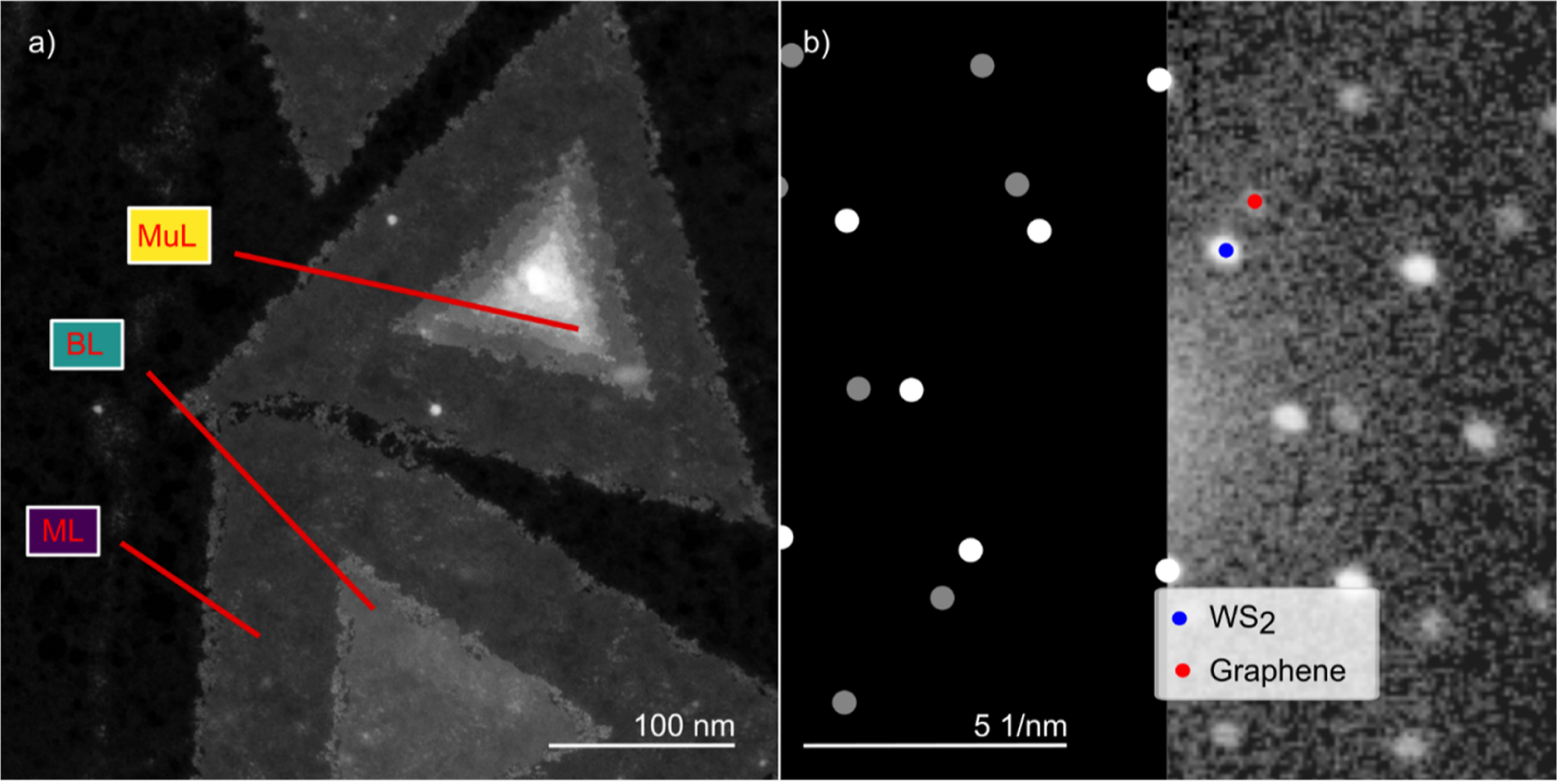
(a) Two WS_2_ flakes on graphene with regions of mono- bi- and multilayered material, which can be distinguished by their brightness. (b) Nanobeam diffraction (NBD) pattern from the ML region. The left part of the image is a schematic continuation of the diffraction pattern to guide the eye. The diffraction pattern shows both WS_2_ and graphene diffractions, providing evidence of the graphene substrate as well as the heteroepitaxial alignment of the WS_2_ to the graphene.

To take potential epitaxial arrangements, which are a result of the growth process applied to synthesize this sample, into account, we investigate the atomic arrangement of the heterostructure in detail. Figure 2a shows the high resolution (HR) images of different regions of interest (ROIs) on the BL, that are marked in Figures S2 and S3a,b subsequently. It should be emphasized that the stacking is continuously changing into different stacking configurations as one moves around the BL region. We hypothesize that this is due to the alignment to the graphene, which brings the lattice constant of the lower WS_2_ layer closer to that of the graphene than the topmost second layer, as schematically depicted in Figure 2b. This may be because the graphene layer introduces strain to the first layer, which diminishes as the second layer grows. This offset results in a lattice mismatch moiré (LMM), which leads to slight, continuous changes in the stacking configuration. We prove this by fitting the positions of all the atoms in the lower and upper WS_2_ layers using Atomap.^42^ This fit shows an increase of 1 pm in the lattice constant of the upper layer compared to the lower layer. However, it reveals no twist between the layers, which rules out the possibility of a classic twist-moiré. Details of this investigation can be found in Supplementary part II. We would like to note that it is not possible to detect the lattice mismatch using the Fourier transformation of real-space images or the diffraction pattern in a TEM investigation since the offset of 1 pm is far below the STEM resolution. However, the mismatch is still observable because the continuous change in the stacking can be observed by the overlay of two only slightly different spatial frequencies (the lattice constants of the two layers), leading to an LMM. To further confirm the LMM, we build a supercell consisting of two WS_2_ layers. The topmost layer is chosen 1 pm larger in lattice constant while keeping the remaining parameters the same for both layers (*i.e.*, no twist, strain, or other distortions). Figure 2a shows a comparison of different regions of interest (ROIs) from the experiment and the abTEM simulation of the same ROIs from the constructed supercell. We observe that the stacking configurations of all ROIs in the experiment align well with the simulations of the constructed supercell. Figure S3 shows the location of the ROIs on the sample. Also fits performed in a region far away from that shown in Figure S3 but on the same flake reveal the same lattice constant mismatch. It is remarkable that also here the entire scan region can be reassembled using a single synthetic supercell, as shown in Figure S4. This underlines the controlled impact the growth from the gas phase has on the structure of the TMD film.

**Figure 2:**
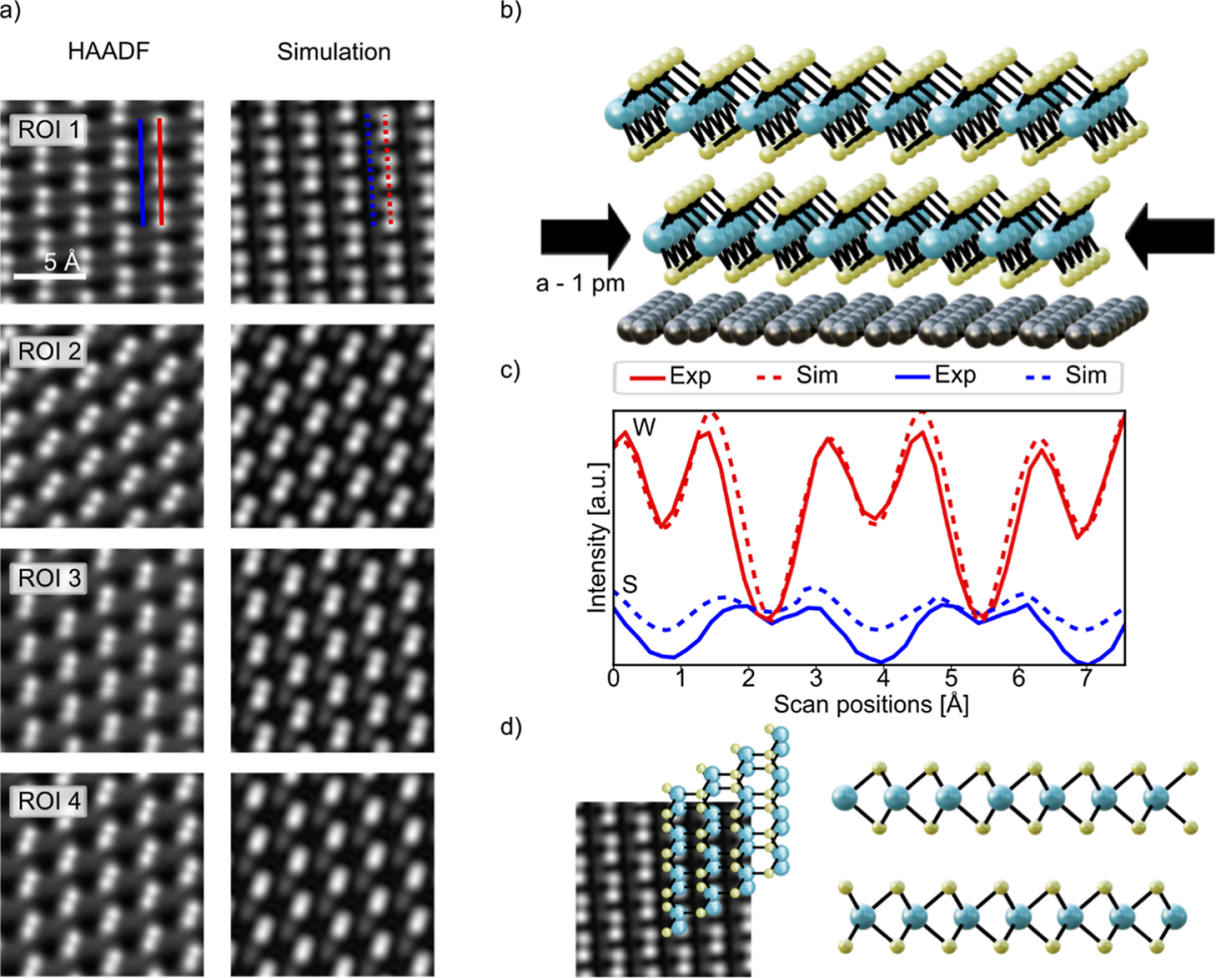
(a) Different regions of the BL material, like marked in Figure S3 (left) in comparison to the STEM-simulation (right). (b) Schematic depiction of the WS_2_ layers on graphene. The lower layer has a reduced in-plane lattice constant by 1 pm compared to the lattice constant of the second layer. For illustrative purposes, the difference in the lattice constants has been exaggerated in the picture. (c) Line scan across W- and S atoms, along the lines depicted in (a) in ROI1. (d) Supercell used for the *ab initio* simulations. The atomic arrangement is matching to the stacking depicted in ROI 1. The supercell is shown from the top- (left) and in side-view (right).

By comparing the intensities and atomic configurations in Figure 2a,c in the line scan of ROI1, one can determine the stacking order within the BL region to be AA-stacking with a slight offset (depicted schematically in Figure 2d), thereby facilitating the later alignment of *ab initio* calculations with this specific stacking configuration. However, we expect this stacking to continuously change into different stacking configurations, when moving to different regions in the BL material, because of the LMM. Nevertheless, we use this stacking configuration for the *ab initio* simulations, since ROI1 is close enough to the line, where the EEL spectrum is recorded.

Figure S2 illustrates the extent of the LMM relative to the BL region of the flake. It includes a representation of the LMM supercell, virtually repeated several times to highlight its periodic structure. As shown, the LMM supercell spans 100.4 nm, approximately matching the overall size of the BL region.

It should be noted that this kind of LMM is only to be expected from an epitaxially grown, but not from an exfoliated sample, where the single layers should have their equilibrium lattice constant. It is, however, very important to know these alignments, which—despite of being extremely little—have a large impact on the exciton optics, as we will show below.

The EEL spectra, presented in Figure 3a, are acquired by performing a line scan across the two flakes discussed in the previous paragraph in the direction shown in Figure 3b. We place an aperture around the forward scattered electron spot, like indicated in Figure S5 in supplementary part III by the red circle, allowing for momentum transfers covering up to 75% of the Brillouin zone. The spectra are presented by smoothing signals from all scan points by Savitzky–Golay,^43^ binning and averaging the scan points that correspond to the specific structures of ML, BL and MuL. To accurately match the signals to the scan position, the clustering tool UMAP^44^ is used. Moreover, the zero-loss peak’s (ZLP) residual tail, originating from additional scattering phenomena, *e.g.* radiation losses, is subtracted by applying a power law model. Additionally, a dedicated scan of the region exclusively containing the graphene substrate is executed. Subsequently, this substrate-specific data is subtracted from each of the spectra, effectively eliminating the contribution from graphene and ensuring a more focused analysis of the WS_2_ spectrum.

**Figure 3:**
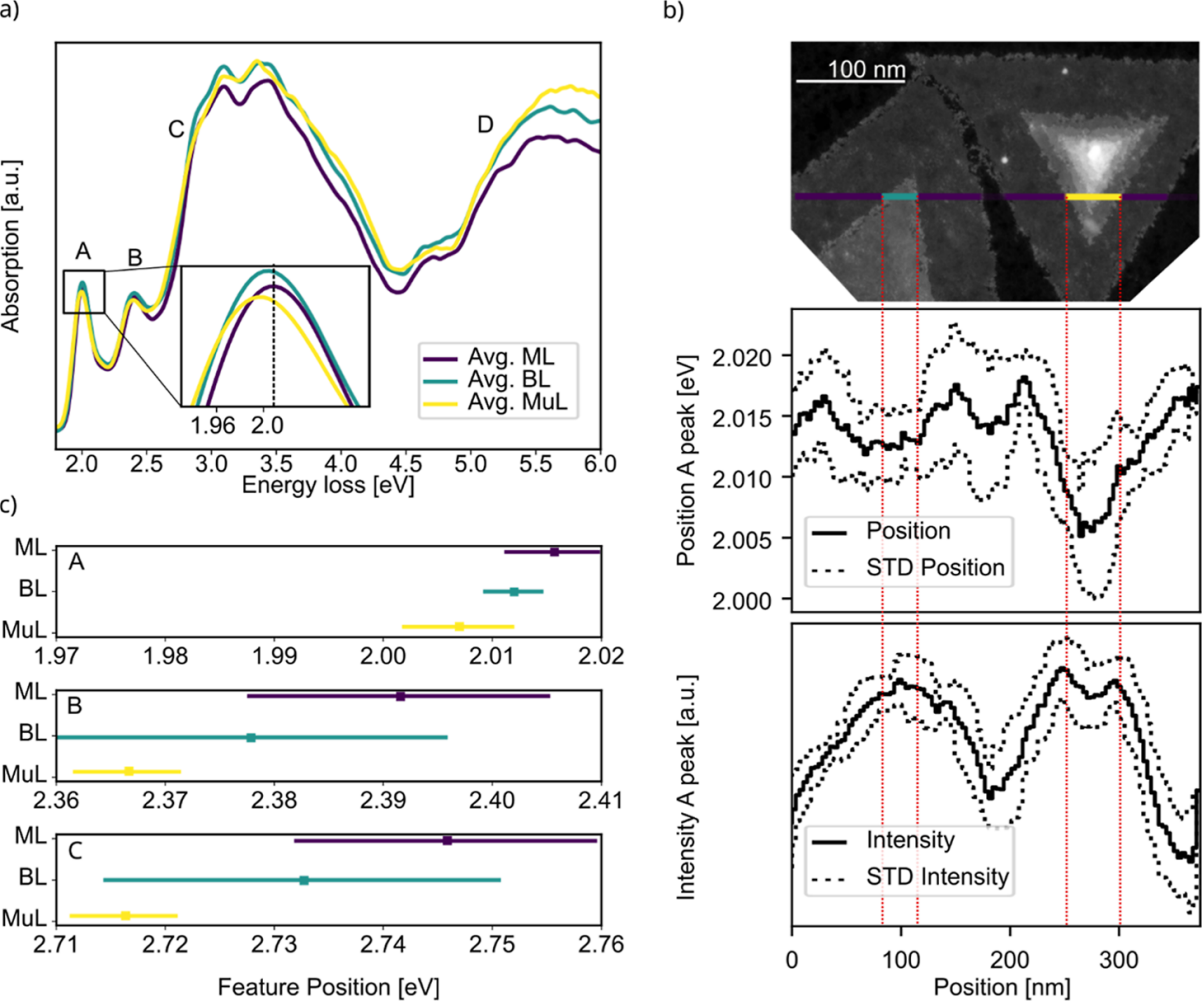
(a) EEL spectrum of the three distinct regions labeled as ML, BL and MuL in Figure 1a. The most prominent features are labeled as A, B, C and D. The inset shows the redshift of the A peak around 2 eV. (b) EELS scan line across the two flakes. In the images beneath the position and the intensity of the A peak is plotted, correlated to the scan position. The position and intensity in Figure 3b are smoothed by a moving average window and the STD values are the standard deviations for each moving window. (c) Peak shifts relative to the peak position of ML region I for the three features A, B and C shown in Figure 3a.

The data set in Figure 3a manifests two prominent excitonic peaks positioned at 2.0 and 2.4 eV, which we assign as A and B, in accordance with prior work measuring optical reflectance.^35^ Additionally, an edge C and a peak D is detected, spanning the energy range of 2.7 to 2.9 eV and 5 to 6 eV, respectively. The inset within Figure 3a highlights the A exciton peak position at different regions ML, BL and MuL, revealing a notable redshift with increasing layer number. The MuL region in our experiment is 4 layers thick, as concluded from its intensity in the HAADF image. The A peak, which corresponds to an optically bright transition is also successfully detected using microphotoluminescence spectroscopy, as shown in Figure S6 in the supplementary part IV. It is observed throughout the entire sample, underlining that our observations are a general property of the heterostructure. To quantitatively assess the spectral changes in the EELS data depending on layer number, an analytical fit to the entire spectrum shown is performed. This fit encompasses three Lorentzian distributions in conjunction with a Fermi function applied to the data within each respective region, thereby facilitating the precise determination of the energy positions of the distinct peaks and the edge C. Further elaboration on this fitting procedure together with the raw spectra can be found in Figure S7 in the supplementary part V. Figure 3c then displays the shifts of the A and B peaks, as well as of the edge C, that are the averages of the fitting results. The error bars correspond to the standard deviation of all fitted positions in the corresponding regions ML, BL and MuL. A redshift is evident for all features with increasing layer number. The redshift is only a few millielectronvolts (meV) for the lower energy features, but it becomes more significant for the higher energy features, reaching a few tens of meV, as seen for feature C. Figure 3b also illustrates the correlation between the redshift of the A-peak, as found by the fit, and structural changes. As the measurement approaches the BL region, the peak position undergoes a redshift of approximately 5 meV, which reverses upon transitioning back to the ML region. By moving toward the MuL region, the redshift becomes even more pronounced. In terms of intensity, a slight increase is observed when transitioning from the ML to the BL region, while a decrease compared to the BL is noted in the MuL region. This behavior may be attributed to the increased material thickness enhancing absorption initially. However, in thicker regions, electronic effects likely modify the oscillator strengths, reducing the absorbance.

To achieve a comprehensive understanding of the experimental results, we now turn to DFT-BSE calculations. We model the regions of ML and BL, respectively, first as freestanding WS_2_ MLs and freestanding stacked BLs, as indicated in Figure 2d, without the graphene substrate. The computed EEL spectrum for zero momentum transfer of the ML (cf. Figure 4a: BSE Δ*q* = 0) shows two prominent excitonic features labeled A and B. Except for a small blueshift of the simulation of 15 meV in comparison to the experiment, these features are consistent with the experimental observations both in terms of the absolute energy, as well as the peak distance between A and B of 0.36 eV due to the spin–orbit coupling^45^ at the *K*-point of the Brillouin zone. The spectral features at higher energies beyond 2.5 eV, namely C and D peaks, also match the experimental observations. However, at energies between 3.25 and 4.2 eV there seems to be a lack of intensity in comparison to the simulation. This can be due to the neglected momentum transfer in the simulation, since the practical implementation of the aperture in the experiment (see Figure S5) allows momentum transfers. Therefore, we also calculated the spectra for small momentum transfers Δ*q* = 0.0121/Å and Δ*q* = 0.0211/Å, that in reciprocal space fall within the (000)-diffraction spot, *i.e.* have a reasonable intensity contributing to the spectrum. These contributions are rather small and decay rapidly with increasing q for contributions in the vicinity of the A and B peak. Energy contributions above 3.25 eV become more pronounced and the cumulative effect of integrating over the entire Brillouin zone may alter the spectrum, providing a potential explanation for the discrepancies between simulation and experiment in the vicinity of 3.25 eV, in agreement with the findings by Hong *et al*.^46^ We note that the simulations in this energy range may be slightly under-converged with respect to the number of empty bands. However, extending the simulations to include additional bands leads to only minor changes in the spectrum up to 5 eV, reinforcing the validity of our argument. We also note that finite q also shifts the features A and B to higher energies, what is also in agreement with Hong *et al*.^46^ Future studies should explore contributions at finite momentum transfers, potentially revealing dark excitons. These excitons are likely detectable with EELS due to the momentum transfer imparted by the electrons.

**Figure 4:**
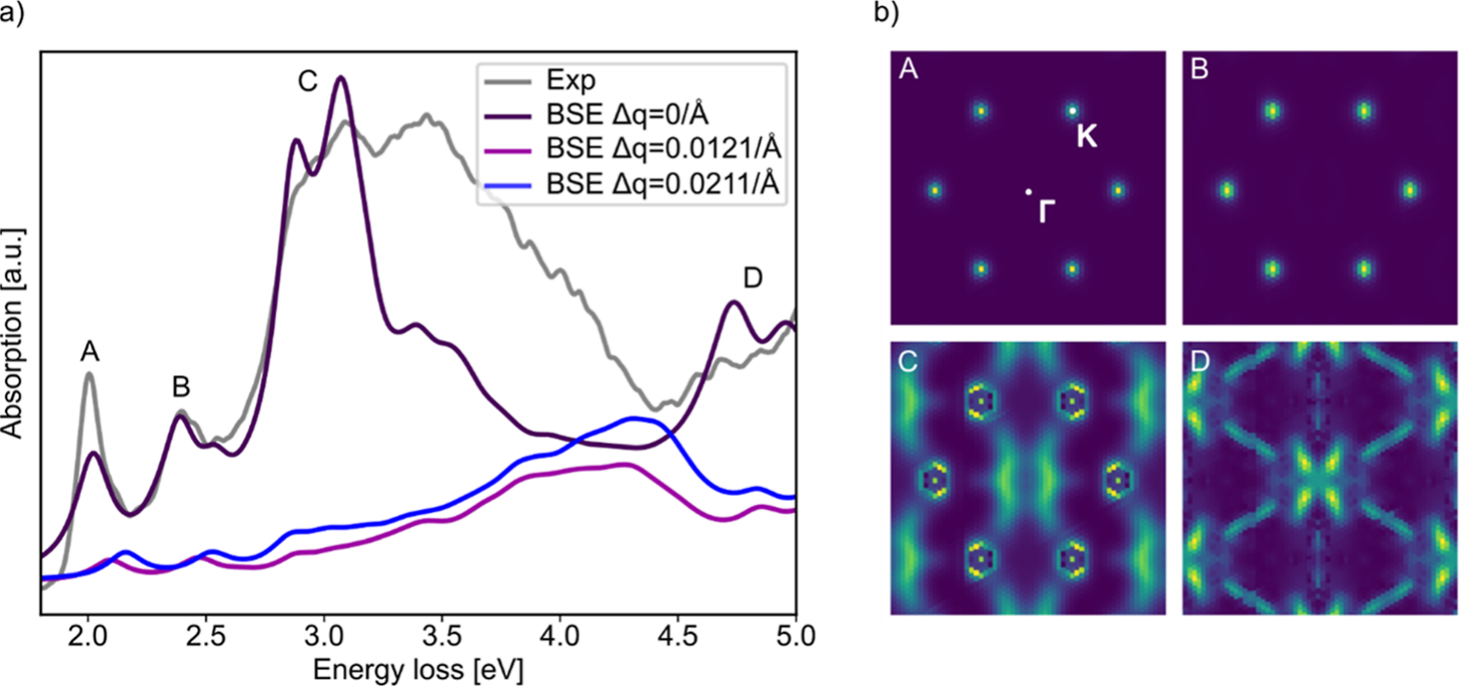
(a) Results of the BSE simulations for ML WS_2_ for different momentum transfers in comparison to the experimental result. (b) Oscillator strengths of the ML BSE simulation in momentum space for the four different peaks indicated by the labels in Figure 4a.

To clarify the origin of the features in the simulation and hence the experiment, we show the oscillator strengths in momentum space in Figure 4b, that are calculated by the eigenvectors of the BSE Hamiltonian, corresponding to the four prominent peaks labeled as A, B, C and D in Figure 4a. A and B originate mainly from excitations at the *K*-point, indicating their association with the spin–orbit split *K*-point excitons already known from literature.^45^ In contrast, the origins of the C excitations appear more multifaceted. Points between Γ and K within the Brillouin zone contribute significantly, which has been attributed to band nesting effects reported in previous studies.^47^ The feature D does not show any contributions from the *K*-point. Instead, it is dominated by contributions centered around the Γ point, suggesting a distinct class of excitations.

We now turn to the dependence of the EEL spectra on the in-plane lattice constant of the WS_2_ layers. Our present EELS measurements reveal a clear redshift of the A exciton as the of number of WS_2_ layers increases. In our system, this shift correlates with a small but measurable lattice expansion of approximately 1 pm in the second WS_2_ layer compared to the first. While previous studies based, *i.e.*, on reflectance spectroscopy,^35,48^ ellipsometry, absorbance^49^ and photoluminescence,^50^ also report redshifts with increasing layer number, these measurements were conducted on exfoliated samples, where strain effects are negligible and the lattice constants are identical across layers. Consequently, those systems are not directly comparable to our epitaxially grown heterostructures, in which strain is introduced *via* lattice alignment to the graphene substrate in a remarkable precision, as explained above.

In contrast to purely optical techniques, our EELS measurements offer nanometer-scale lateral resolution, allowing a direct correlation between excitonic energy shifts and local structural features. Specifically, we observe that the red-shifted A exciton coincides with bilayer regions exhibiting an LMM and an AA-like stacking configuration with a small lateral offset. This suggests that the observed spectral shift originates not primarily from quantum confinement effects, but from local variations in the lattice constant induced during growth.

To further explore this mechanism, we analyze the band structures of WS_2_ monolayers and bilayers, as shown in Figure 5a, including a comparison to a strained bilayer that approximates the experimental lattice mismatch. As the system transitions from ML to BL and MuL configurations, interlayer coupling modifies both the dielectric environment and the electronic structure. To approximate the experimental conditions, we introduce uniform strain into both layers of the of the bilayer structure, mimicking the effect of lattice mismatch. A more accurate model, where only the bottom layer is strained to reflect epitaxial alignment to graphene, would require large supercells (as the LMM has a lattice constant of more than 100 nm, see Figure S2) and is computationally intractable for full DFT–BSE calculations. Despite this simplification, the model captures the essential impact of strain on the electronic structure and the impact of the screening environment.

**Figure 5:**
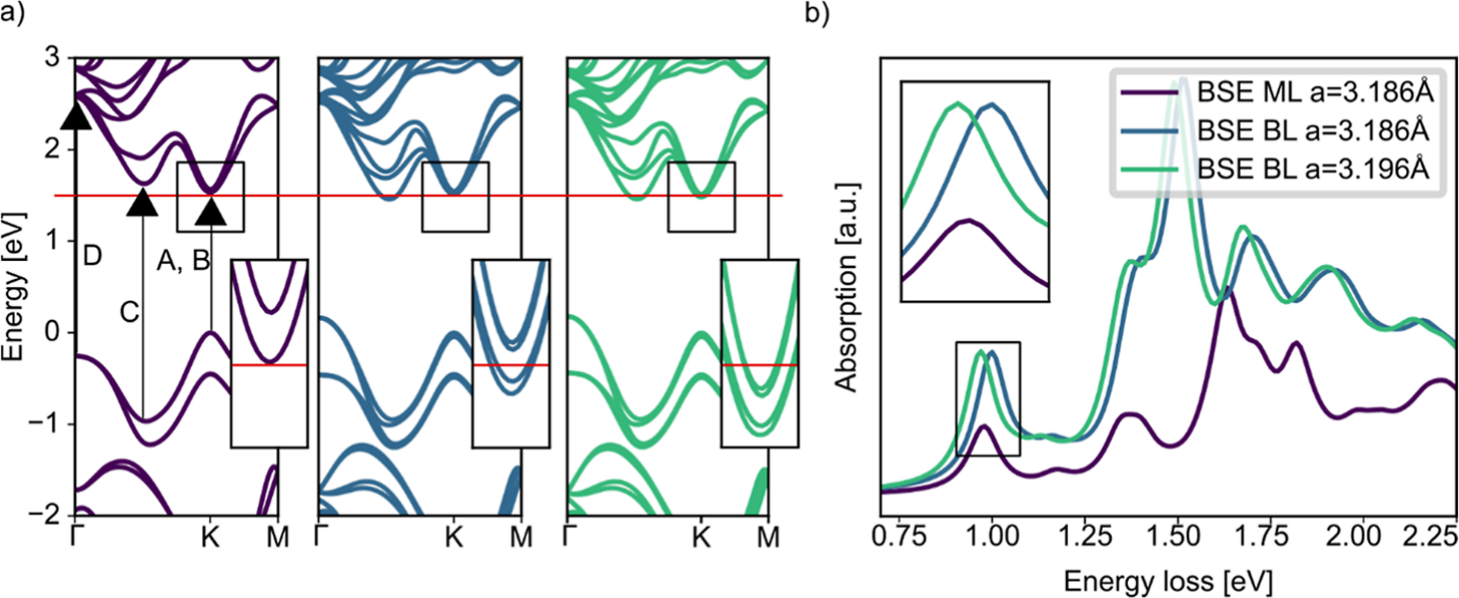
(a) DFT band structure of ML WS_2_ (left), unstrained BL (middle) and strained BL (right). The arrows show the transitions in momentum space, where the prominent features A, B, C and D appear. A splitting of the bands is visible at the *K*-point in the BL figures, leading to a reduction of the band gap. (b) BSE results for the three cases ML, BL and strained BL.

The dominant optical transitions, illustrated in Figure 4a, that are contributing to the A, B, C and D excitonic peaks are indicated by arrows in the band structure shown in Figure 5a. Interlayer interactions in the bilayer system lift the degeneracy of electronic bands, resulting in deviations from the ML band structure and the appearance of band splittings, as highlighted in the insets of Figure 5a. At the *K*-point, we observe a small splitting of 53 meV and a band gap reduction of 23 meV for the unstrained bilayer, primarily due to the reduced quantum confinement. In contrast, more pronounced band splitting occurs between Γ and K.

When strain is introduced, the BL bandgap is further reduced by 43 meV relative to the monolayer, leading to a significant redshift in the excitonic spectrum. This is evident in the simulated spectra shown in Figure 5b: while the unstrained BL spectrum exhibits a slight blueshift compared to the ML, the strained BL shows a clear redshift, in quantitative agreement with our experimental observations. The blueshift in the unstrained BL arises because the additional dielectric screening reduces the exciton binding energy, which more than compensates for the moderate band gap reduction. In the strained case, however, the larger bandgap reduction dominates, resulting in a net redshift. This highlights the critical role of lattice strain induced by epitaxial alignment to the graphene substrate. Supporting this interpretation, prior measurements on WS_2_ bilayers grown without a graphene substrate report a blueshift of the A exciton relative to the monolayer.^18^ We note that this overall mechanism does not dominate the behavior of the C exciton. In that case, the stronger gap reduction between Γ and K results in a redshift of the C peak already in the unstrained bilayer, which is further enhanced in the strained configuration. This trend is also observed in our EELS measurements, where the C feature exhibits a more pronounced redshift than the A exciton.

Understanding the influence of the graphene substrate on the electronic structure of WS_2_ is crucial for future device applications.^11,12,19–22^ Its presence contributes to the experimentally observed EEL spectra, either indirectly through dielectric effects,^51^ directly by modifying the electronic structure, or by altering the WS_2_ lattice constant *via* heteroepitaxial alignment, as discussed above. Graphene has been shown to cause a redshift of the A exciton peak in WS_2_.^52^ In our case, however, the effects of dielectric screening and strain cannot be disentangled experimentally. An exfoliated or freestanding WS_2_ layer would not experience strain from lattice alignment, whereas in our epitaxial system, the lattice constant is modified by the underlying graphene. Both scenarios lead to different A exciton positions, but for fundamentally different reasons. As a result, one can either probe the effect of dielectric screening and quantum confinement using exfoliated samples or—as in the present work—study the impact of strain induced by epitaxial alignment. A clean separation of these contributions is not possible within a single measurement.

To further assess the impact of lattice strain, Figure 6a shows BSE-calculated EEL spectra for ML and BL WS_2_ over a range of lattice constants from 3.05 Å to 3.25 Å. A direct simulation of the experimentally observed BL structure, where only the upper layer exhibits a lattice constant that differs by 1 pm compared to that of the lower one, would require a supercell exceeding 100 nm in size and is thus computationally prohibitive. Instead, both layers were modeled with modified lattice constants. This approximation captures the essential physics of dielectric screening and quantum confinement, while still reflecting the impact of lattice variation. To further reduce computational cost, these simulations were carried out using the PBE functional. As expected, the resulting spectra are rigidly shifted to lower energies due to the well-known band gap underestimation of PBE. However, as shown in Figure 6b, the qualitative trends remain consistent: across all lattice constants, the A exciton peak exhibits a blueshift when transitioning from ML to BL. As shown in the inset of Figure 6b, a lattice mismatch of 0.6 pm is required to compensate exactly for the blueshift caused by the screening effect of the second layer and recover the A-peak energy of the unstrained ML. Thus, BLs with lattice constants exceeding 0.6 pm relative to the ML—consistent with our experimental measurements—result in a net redshift of the A-exciton. These findings corroborate our experimental observations and further support the conclusion that the dominant contribution to the exciton redshift arises from strain-induced band gap renormalization, rather than from quantum confinement or dielectric screening alone.

**Figure 6:**
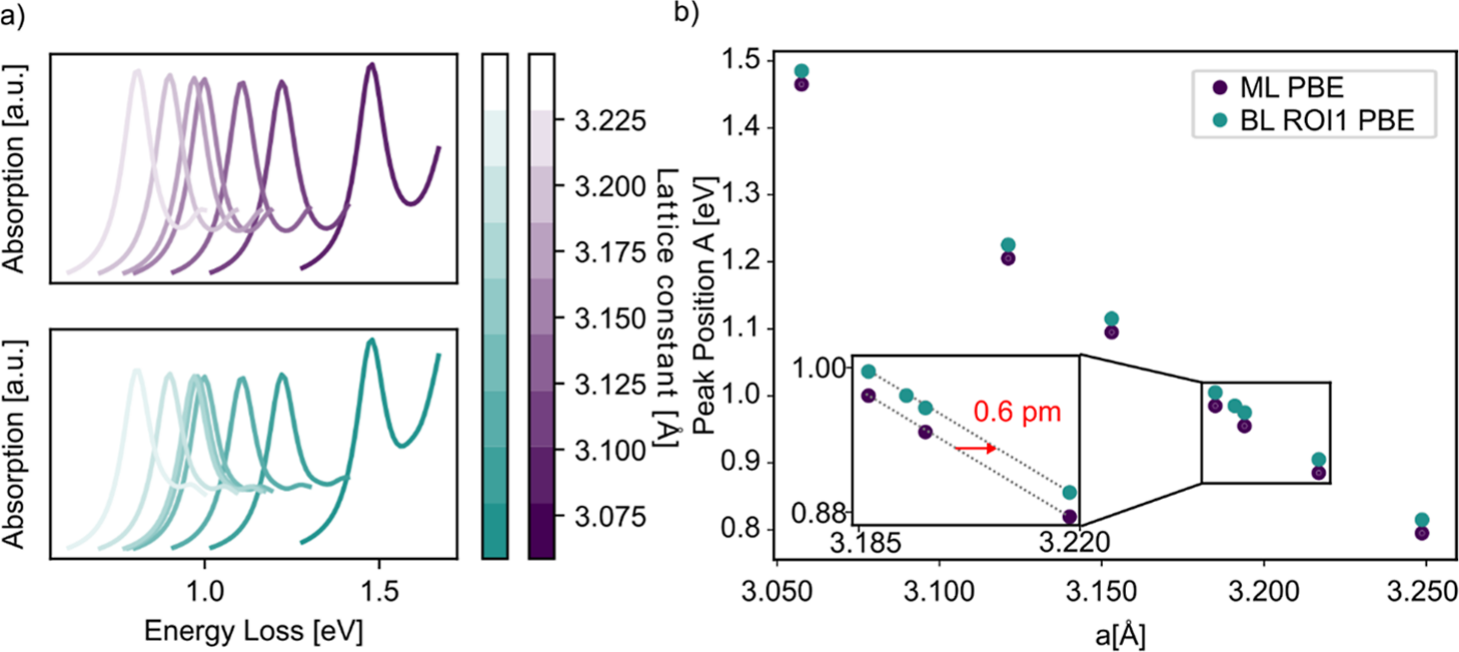
(a) BSE results of ML (upper panel) and BL (lower panel) WS_2_ for different lattice constants, focusing only on the region surrounding the A peak. (b) Position of the A peak from the simulations shown in (a) plotted against their corresponding lattice constant. The inset shows, that a lattice constant mismatch of more than 0.6 pm is enough to get a redshift from the ML to the BL A-peak of the spectrum.

## Conclusion

Using nanometer-resolved electron energy loss spectroscopy (EELS) combined with *ab initio* simulations, we investigated the excitonic response of epitaxially grown WS_2_ on graphene, a heterostructure of high technological relevance. Our measurements reveal a redshift of the K-valley A and B excitons in the bilayer configuration, which we attribute to minute changes in the lattice constant between the WS_2_ layers induced by epitaxial constraints. This structural modification is directly observed in high-resolution scanning transmission electron microscopy (STEM) as a lattice mismatch moiré (LMM) pattern. By mapping the measured EEL spectral features to specific transitions in the WS_2_ band structure, we demonstrate that the observed redshift originates predominantly from strain-induced changes in the lattice constant, rather than from quantum confinement. *Ab initio* simulations confirm that this effect dominates for the A exciton and becomes even more pronounced for features above 2.5 eV, particularly for the C exciton, which arises from states between Γ and K. This feature is highly sensitive to stacking and interlayer interactions.

Our investigations establish a direct correlation between local structural variations and excitonic properties across a wide energy range. This highlights the critical role of epitaxial strain and heterointerface design, offering new insights toward tailoring layered materials for device applications. As the WS_2_/graphene heterostructure is grown *via* state-of-the-art and up-scalable gas phase processes, our findings are directly applicable to future device integration in the semiconductor industry.^11,12,19–22^

## Methods

The WS_2_-graphene heterostructure under study is synthesized using MOCVD in a 19 × 2 in. Close Coupled Showerhead AIXTRON MOCVD reactor. The process is conducted on *c*-plane sapphire substrates, which possess a 0.2° off-cut toward the m-plane. The fabrication of the heterostructure started with the deposition of graphene on the sapphire substrate. This is achieved using methane as a precursor at a temperature of 1400 °C, incorporating a surface pretreatment step involving hydrogen.^53^ Following this, WS_2_ nucleation is carried out on the graphene-coated sapphire template. This process utilizes tungsten hexacarbonyl (W­(CO)_6_) and ditertiarybutylsulfide (DTBS) as precursors, with a growth temperature set at 700 °C and a growth duration of 1800 s, as described in before.^54,55^

Subsequently, an etchant-free transfer technique is employed to relocate the specimen onto a lacey carbon STEM grid. This results in a free-standing sample spanning the holes between the carbon support (as visually depicted in Figure 7a). In the initial stage of this transfer process, polymethyl-methacrylate (PMMA) is spin-coated onto the sapphire substrate. Subsequently, the specimen is immersed in water heated to a temperature of 80 °C. This immersion results in the intercalation of water between the thin film and the substrate, facilitating the film’s detachment from the substrate. The 2D film is then collected onto the TEM grid and the PMMA is subsequently dissolved using dichloromethane. The process is schematically depicted in Figure S8 in the supplementary part VI.

**Figure 7:**
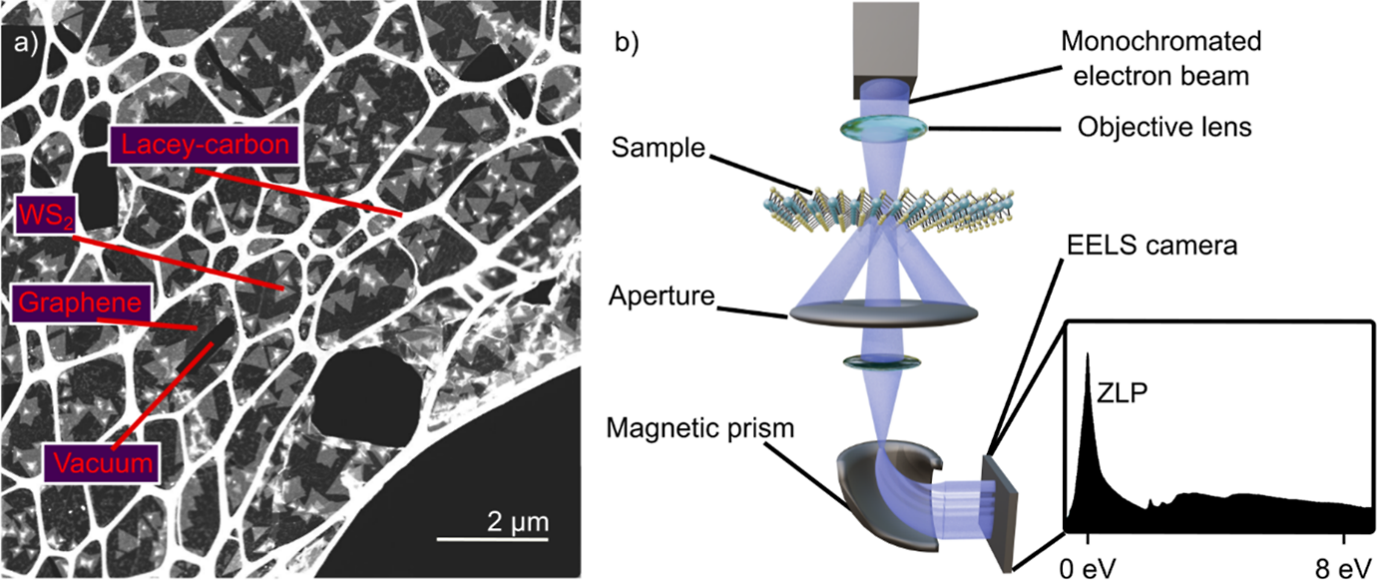
(a) Overview of the freestanding WS_2_ on graphene sample on the lacey-carbon grid. The latter is the spider-web-like structure that serves as a support for the thin 2D material. The triangles are the WS_2_ flakes laying on top of a graphene layer, that gives slightly more contrast in comparison to the vacuum in the bottom right of the image. (b) Schematic of a STEM-EELS setup. The EEL spectrum shows a zero-loss peak (ZLP) and the excitonic features.

HAADF images are taken using a double aberration-corrected JEOL 2200FS STEM operated at 80 kV with a semiconvergence angle of 21 mrad. In HAADF mode high-angle Rutherford-like scattered electrons are detected. Notably, the scattering cross section of these electrons is proportional to the atomic number (Z) of the sample material, following an empirical relationship of between Z^1.5^ and Z^2^ depending on the detector angle. This relation forecasts higher contrast for the W atoms in comparison to the S atoms as well as an increased brightness for increasing number of layers. The EELS-data is collected using a monochromated JEOL JEM-ARM200F NEOARM STEM operated at 60 kV applying a double-Wien filtered monochromator limiting the energy width of the probe to 35 meV. A probe semiconvergence angle of 1 mrad is chosen resulting in a spot size of 6 nm. Using an aperture in the diffraction plane a momentum transfer can be selected. We choose an aperture of the size of 300 μm indicated in Figure S5 by the red circle. A Gatan GIF Continuum HR, an energy filter followed by an EELS camera, is then used to record the spectrum. The EELS dispersion is 5 meV/channel. The setup is schematically depicted in Figure 7b. The EELS data is evaluated using the Hyperspy python package.^56^

STEM image simulations are done using the abTEM python package^57^ adjusting the microscope parameter to match the one used in the experiment. Additionally, 50 frozen phonon configurations are included in the simulation to account for the finite temperature of the sample.

In order to understand the origin of the experimentally observed excitonic features, we perform density functional theory (DFT) calculations as implemented in the GPAW-package^58–60^ and the Atomic Simulation Environment (ASE).^61^ The electron–ion interaction is described *via* projector augmented wave pseudopotentials^62^ and the Perdew–Burke–Ernzerhof (PBE)^63^ exchange–correlation functional. For the bilayer system, van-der-Waals interactions between layers are accounted for during geometry optimization using the DFT-D3 dispersion correction^64,65^ in conjunction with Becke–Johnson damping.^66^ To address the known underestimation of the bandgap in PBE, we perform self-consistent hybrid functional calculations using PBE0.^67,68^ In Figure S9 we compare band structures calculated with single-shot G_0_W_0_ and PBE0. While both approaches yield similar bandgaps at the *K* point, PBE0 predicts a higher conduction band dispersion along the Γ–K direction, which leads to significantly better agreement with the experimental position of the C-feature in the EEL spectra. The improved agreement reflects the importance of self-consistency in capturing the correct conduction band shape, as also noted in prior work on self-consistent GW calculations for related TMD systems.^69^ Spin–orbit coupling is included via nonself-consistent diagonalization on top of the self-consistent PBE0 electronic structure, which then serves as input for EEL spectra computations. Excitonic effects are incorporated at the level of many-body perturbation theory by solving the Bethe–Salpeter equation (BSE) based on the PBE0 reference. In the BSE calculations, the Brillouin zone integration is performed on a Monkhorst–Pack mesh corresponding to 30 × 30 × 1 *k*-points in the primitive WS_2_ unit-cell. To eliminate spurious Coulomb interactions between periodic images in the out-of-plane direction, a 2D truncation scheme^70,71^ is employed. We converge the EEL spectra with respect to the number of occupied and empty states entering the BSE calculations up to an energy of 3.25 eV, achieved by including 6 topmost valence bands (VB) and 6 lowest conduction bands (CB) of the WS_2_ ML system (cf. Figure S10a). We also converged it with respect to the number of *k*-points used in the BSE calculation (cf. Figure S10b). In the comparison between ML and BL systems, we consider only the 4 highest VBs and the 4 lowest CBs for the ML. For the BL system, we doubled this number to 8 VBs and 8 CBs to account for band splitting due to interlayer interactions, ensuring a fair comparison between both systems. This choice ensures consistent and well-converged spectra within the energy range of interest.

## Supplementary Material





## Data Availability

Parts of this research have been previously published on a preprint server^72^ and can be accessed by the following link: http://arxiv.org/pdf/2402.13020.pdf.

## Notes

CIF-Files (ZIP)
The following paragraphs, which appeared chronologically in this work: (I) mapping the graphene orientation, (II) mapping the LMM with atomap, (III) placement of aperture, (IV) PL result, (V) fitting procedure, (VI) methods (PDF)
