# Supplementary material for: Excitons in Epitaxially Grown WS2 on Graphene: A Nanometer-Resolved Electron Energy Loss Spectroscopy and Density Functional Theory Study
Source: ACS Nano. 2025 Dec 11;19(50):42107–17. doi: 10.1021/acsnano.5c11994 (PMC12752690; doi:10.1021/acsnano.5c11994)
Supplement: Supplementary file 2 [file nn5c11994_si_002.pdf]

Supplementary information: Excitons in Epitaxially Grown WS_2 on Graphene: A Nanometer-Resolved Electron Energy Loss Spectroscopy and Density Functional Theory Study

Max Bergmann[1,2]*, Jürgen Belz[1,2], Oliver Maßmeyer[1,2], Badrosadat Ojaghi Dogahe[1,2], Robin Günkel[1,2], Johannes Glowatzki[1,2], Andreas Beyer[1,2], Ivan Solovev[3], Jens-Christian Drawer[3], Martin Esmann[3], Sergej Pasko[4], Simonas Krotkus[4], Michael Heuken[4], Stefan Wippermann[1,2] and Kerstin Volz[1,2]*

[1]mar.quest | Marburg Center for Quantum Materials and Sustainable Technologies, Philipps-Universität Marburg, 35032 Marburg, Germany

[2]Department of Physics, Philipps-Universität Marburg, Hans Meerwein Str. 6, 35032 Marburg, Germany

[3] Institute of Physics, Carl von Ossietzky University of Oldenburg, 26129 Oldenburg, Germany

[4] AIXTRON SE, 52134 Herzogenrath, Germany

** Corresponding authors:*

max.bergmann@physik.uni-marburg.de

kerstin.volz@physik.uni-marburg.de

Supplementary information I: Mapping the graphene orientation.

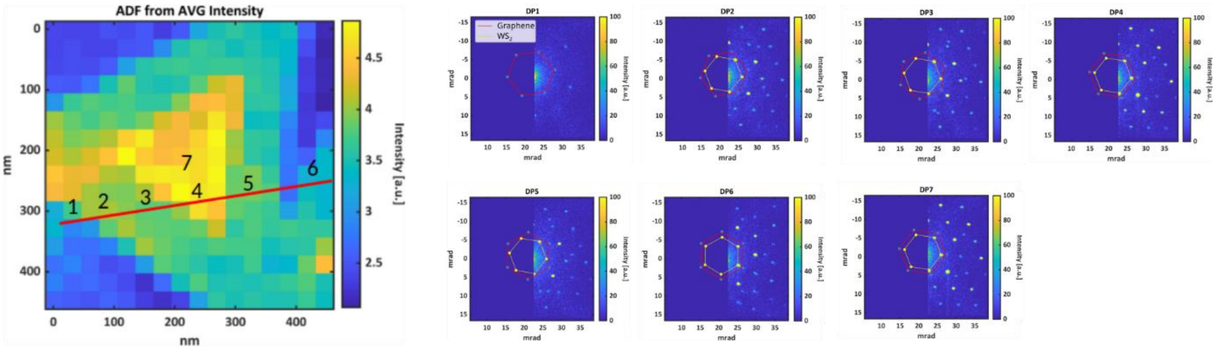

Figure S1: Alignment of the graphene beneath the WS_2 on different scan positions. In the vicinity of the nucleation, namely DP4 and DP7, the WS_2 is clearly aligned to the graphene. This also holds for the adjacent flake, like shown in DP6. The left part of each image was continued synthetically to guide the eye. Moreover, the first order diffraction spots for graphene as well as WS_2 are connected in a honeycomb to facilitate the recognition of the alignment of the two materials.

The general grain size of the graphene flakes varies around 100 nm and shows no preferred orientation to the sapphire. However, this does not apply to the orientation relationship between WS_2 and graphene. Maßmeyer *et al.*.[1] reported that WS_2 tends to grow aligned with the graphene lattice. The nano beam diffraction dataset in Fig. S1 shows that at the center of the flake - where nucleation begins and the BL is located - the growth follows the graphene orientation, consistent with Fig. 1b.

Supplementary information II: Mapping the LMM with atomap

High-resolution images of the BL region, such as Fig. 2a, reveal a spatial alteration in the stacking configuration. This raises the question of whether this alteration is due to a relative twist of the two WS_2 layers or a difference in their lattice constants. Both possibilities produce a moiré-like structure. However, neither possibility is observed by diffraction or an FFT of the image. Therefore, we use the Python-based software package atomap to map all atom positions, which allows us to separate the two layers and determine the moiré type and respective lattice constant. Using the obtained lattice constant, one can create an ASE supercell for the entire region. Fig. S2 provides an overview of two main regions we investigate with the STEM and from which the ROIs in Fig. 2a are taken. Using the synthetic supercell of this region, one can perform a STEM simulation with abTEM, which is also shown in Fig. 2a. We also apply this procedure to the second region depicted in Fig. S2, as shown in Fig. S4. Additionally in Fig. S2 the size of the LMM unit cell is shown in red and is repeated nine times. The unit cell, with 100.4 nm, is roughly the size of the BL flake.

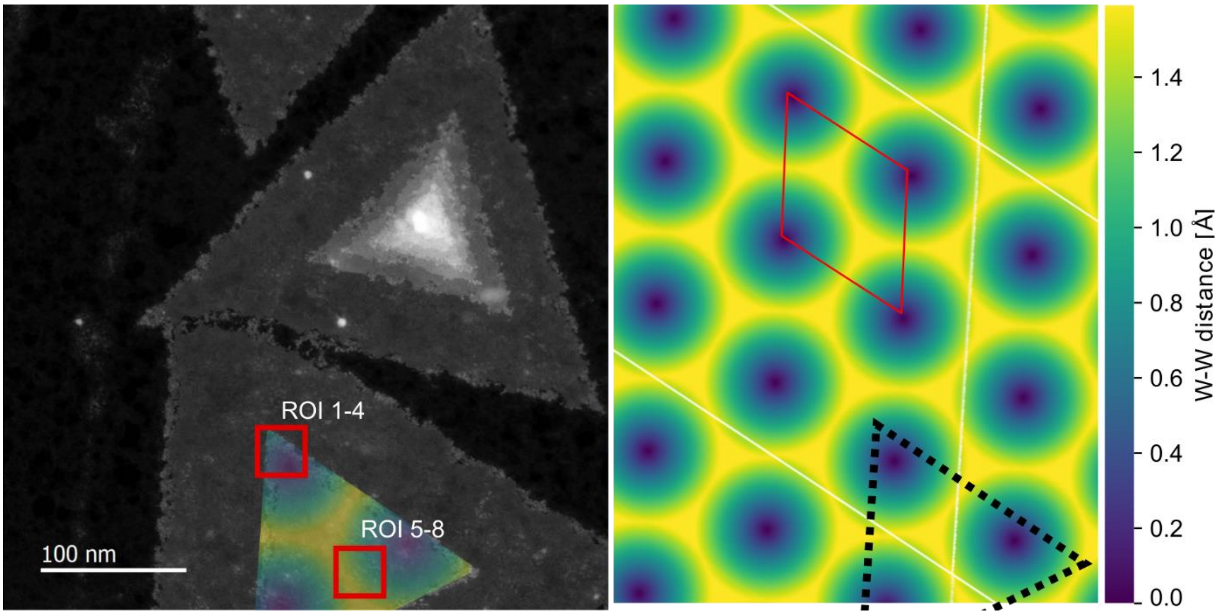

Figure S2: The left image shows an overview of the flake highlighting two main regions we investigate with the STEM. In a transparent overlay the LMM is shown to illustrate the scale of the LMM pattern compared to the flake. The color represents the lateral distance between each W atom in the lower layer and the nearest W atom in the upper layer. The right image shows the virtual repetition of the LMM as it would appear if extended infinitely. One LMM unit cell is highlighted in red, with the dotted region marking the BL observed in the right image. The lattice constant of the LMM is slightly above 100 nm.

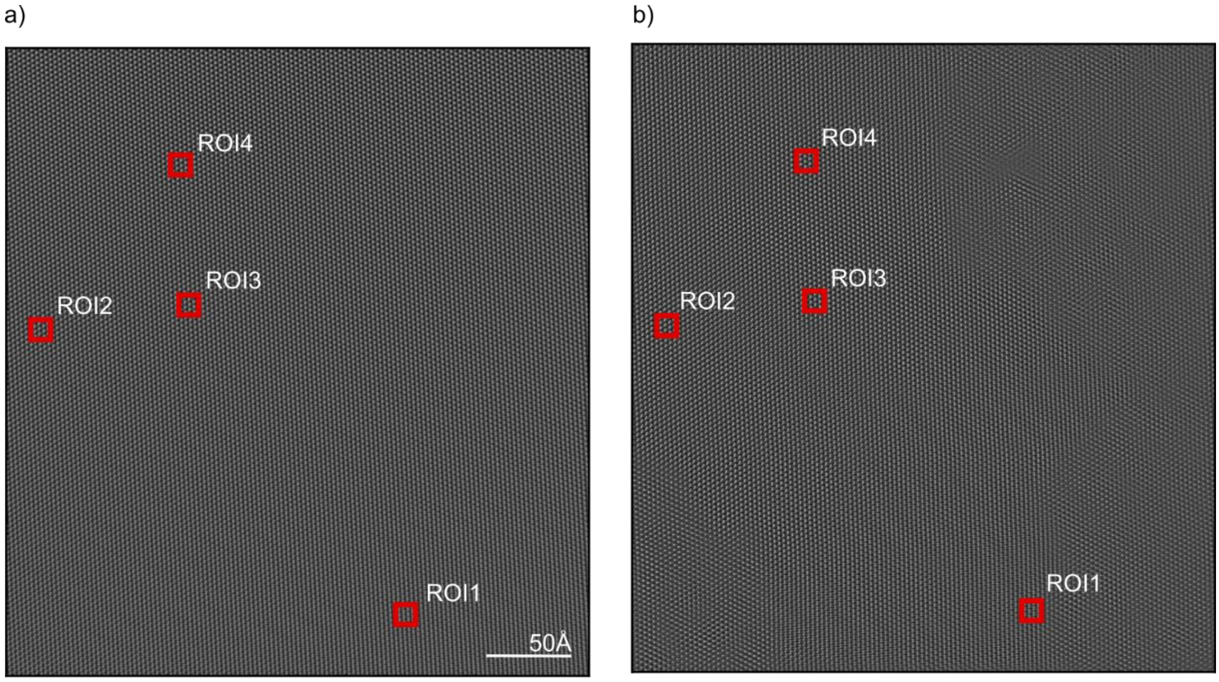

Figure S3: a) Synthetic supercell built by ASE together with the ROIs 1-4 shown in the main text. **b)** Image recorded by STEM after FFT-filtering.

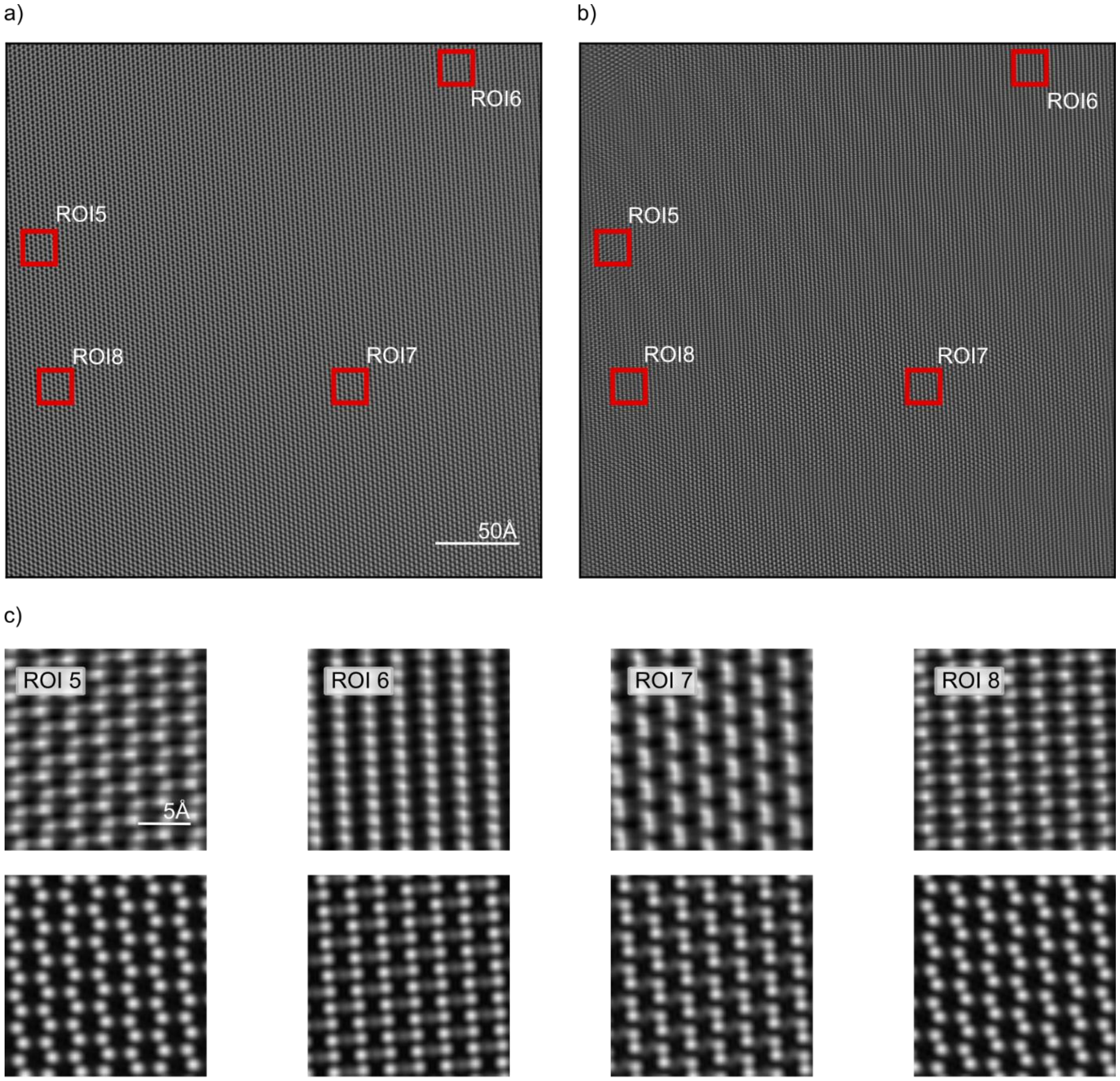

Figure S4: a) Synthetic supercell build by ASE of the second region investigated. **b)** Image recorded by STEM after FFT-filtering. **c)** ROIs 5-8, as marked in a) and b). Upper panels show the STEM measurement, lower panels show the abTEM simulation.

Supplementary information III: Placement of aperture

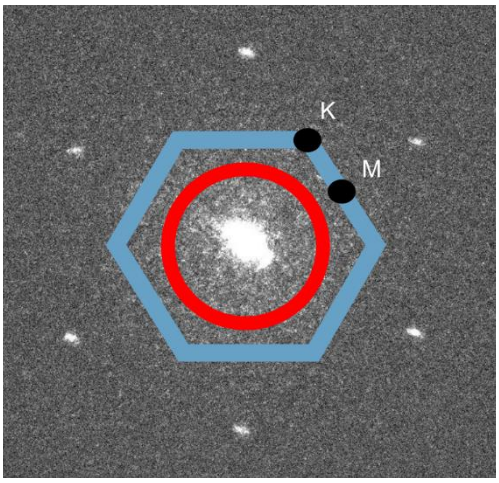

Figure S5: Image of the diffraction plane as obtained in the experiment. The red circle indicates how the aperture is set in comparison to the Brillouin zone, indicated by the blue hexagon.

Supplementary information IV: PL result

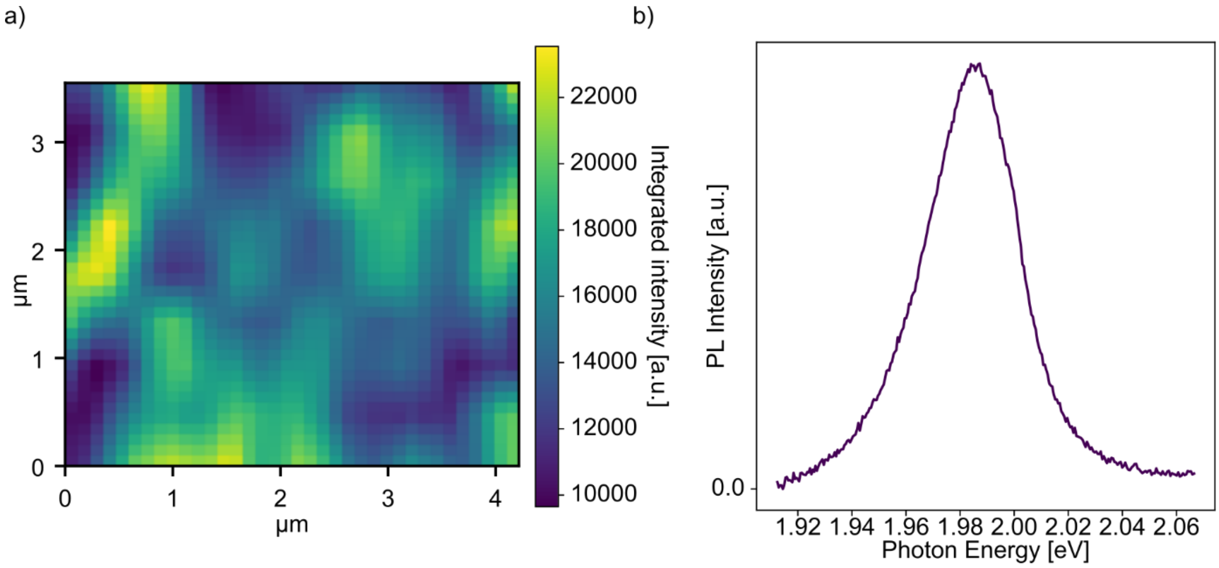

Figure S6: a) The PL map of a random sample region reveals different PL intensities of the A peak depending on the number of flakes in the given scan spot. **b)** Average PL spectrum of the map in a). One obtains a strong peak at 1.985 eV, which fits to the A peak obtained in the EELS study.

Supplementary information V: Fitting procedure

The fitting procedure includes three Lorentzian peaks, labeled A, B and B'. Additionally, the edge C was fitted by a Fermi-distribution. The resulting fit can be seen in Fig. S7a and the fit parameters in Fig. S7c.

Lorentzian:

$$L(x) = \frac{A}{\pi} \frac{\Gamma}{(\mathrm{x} - \mathrm{x}_0)^2 + \Gamma^2}$$

Fermi-distribution:

$$F(x) = \frac{\mathrm{A}}{e^{-\mu(x - x_0)} + 1}$$

a) b)

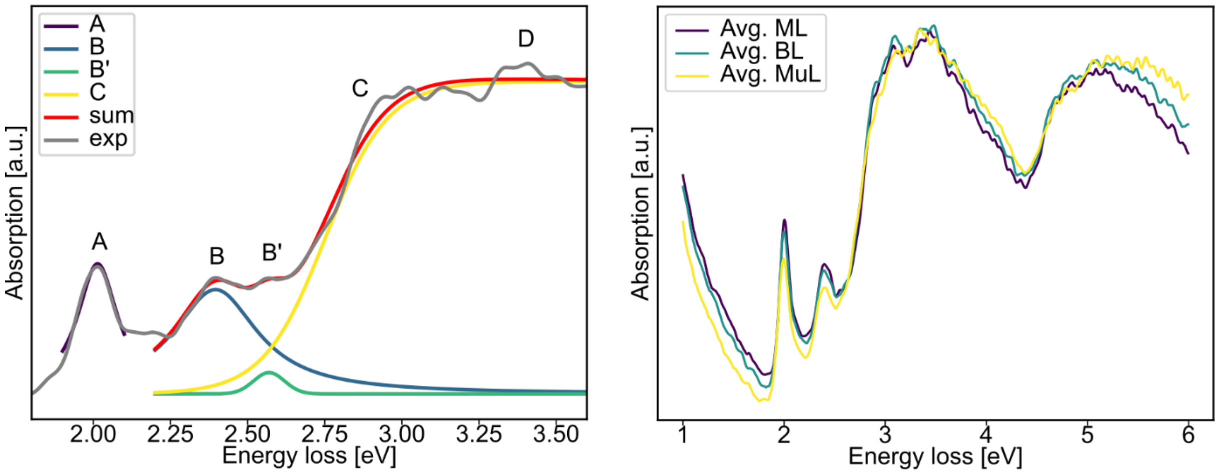

c)

Parameter/#Layer	A	B	B'
x_0[eV]			
ML	2.016	2.392	2.569
BL	2.012	2.377	2.537
MuL	2.007	2.366	2.5
A [eV]			
ML	224948.794	386171.143	14360.236
BL	249573.666	395786.708	24228.649
MuL	256650.566	328668.435	6668.561
Γ [eV]			
ML	0.079	0.159	0.054
BL	0.085	0.163	0.064
MuL	0.094	0.153	0.062

	C
x_0[eV]	
ML	2.746
BL	2.732
MuL	2.716
A [eV]	
ML	2314350.346
BL	2433786.685
MuL	2458436.123
μ [eV]	
ML	0.106
BL	0.109
MuL	0.134

Figure S7: Fitting procedure. a) Exemplaric fit of one of the EEL spectra in the monolayer region. Three Lorentzian peaks (A, B, B') in addition to one fermi function (C) have been found to fit the spectrum. b) Raw average spectra of the ML, BL and MuL region without savitzky-golay filtering or background subtraction. c) Average parameters of the different features denoted in a) in the three different regions ML, BL and MuL as shown in Fig. 1a.

Supplementary information VI: Methods

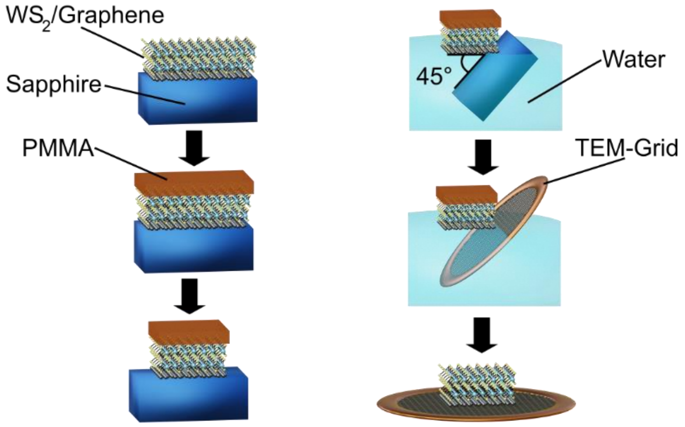

Figure S8: PMMA transfer.

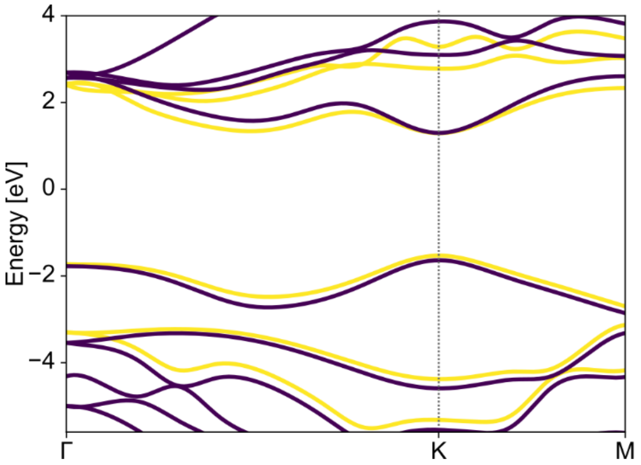

Figure S9: Comparison between the band structure obtained using DFT-PBE0 (purple) and the result obtained with the GW approach (yellow) without spin-orbit coupling. A slight difference in the band gap at K is visible. Also, we note the strong difference at points between Γ and K.

Fig. S9 compares the band structures obtained from self-consistent DFT-PBE0 and single-shot G_0W_0 calculations. While both methods yield a similar bandgap at the K point, notable differences appear along the Γ-K direction: the PBE0 conduction band lies systematically higher between Γ and K, resulting in a larger energy separation relevant for transitions contributing to the C-feature in the BSE spectrum. As a consequence, the C-feature appears at higher energies when using PBE0, in significantly better agreement with the experimental EELS data. This observation is consistent with the findings by H. Shi *et al.*[2], who showed that fully self-consistent GW ($scGW_0$) calculations enhance the conduction band dispersion between Γ and K more than single-shot GW or PBE. We therefore conclude that self-consistency is essential to accurately capture the conduction band shape in this region. Our self-consistent PBE0 results reproduce the qualitative trends reported by Shi *et al.* and provide a computationally efficient alternative to $scGW_0$, especially for complex multilayer systems.

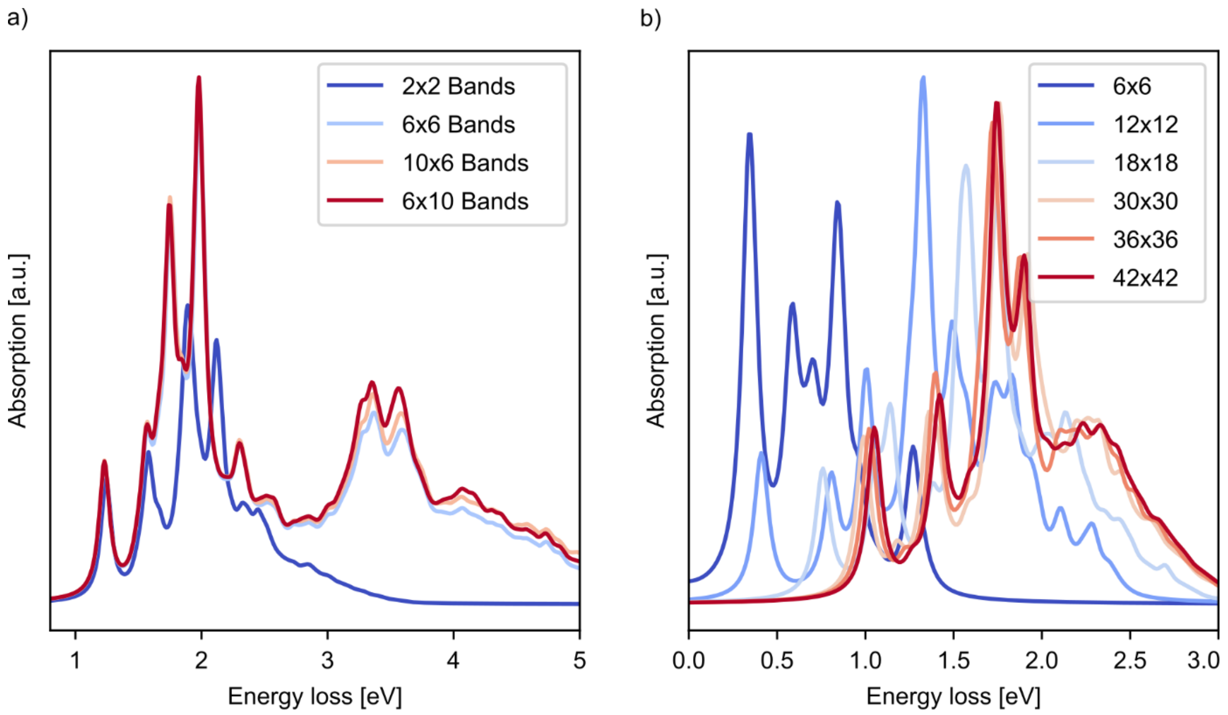

Figure S10: a) Convergence of the BSE calculations with respect to the numbers of valence- and conduction-bands (c-v) included. **b)** Convergence of the BSE calculations with respect to the number of k-points.

Fig. S10a shows the convergence of the BSE calculations with respect to the number of valence and conduction bands included. The 2-2 spectrum is clearly not converged; however, the 6-6 configuration seems sufficient up to 3.25 eV. Minimal corrections are observed when increasing the number of bands to 10-6 or 6-10 above 3.25 eV. Fig. S10b shows the convergence of the BSE calculations with respect to the number of k-points entering the spectrum. All spectra below 30x30 k-points are strongly underconverged, however spectra above 30x30 k-points still yield small corrections to the spectrum, but we stick to 30x30 k-points in all of our simulations to keep computational times feasible.

References

1. Maßmeyer, O.; Belz, J.; Dogahe, B. O.; Widemann, M.; Günkel, R.; Glowatzki, J.; Bergmann, M.; Pasko, S.; Krotkus, S.; Heuken, M.; Beyer, A.; Volz, K. A Small Step for Epitaxy, a Large Step Toward Twist Angle Control in 2D Heterostructures. *Adv. Mater. Interfaces* **2024,** *11*, 2400158.

2. Shi, H.; Pan, H.; Zhang, Y.-W.; Yakobson, B. I. Quasiparticle band structures and optical properties of strained monolayer MoS 2 and WS 2. *Phys. Rev. B* **2013,** *87*, 155304.
